# Antiangiogenic and antiapoptotic effects of green-synthesized zinc oxide nanoparticles using *Sargassum muticum* algae extraction

**DOI:** 10.1186/s12645-018-0037-5

**Published:** 2018-04-02

**Authors:** Zahra Sanaeimehr, Iraj Javadi, Farideh Namvar

**Affiliations:** 1 Department of Toxicology, Faculty of Pharmacy, Shahreza Branch, Islamic Azad University, Shahreza, Isfahan Iran; 2 Departments of Medicine & AMP; Applied Biology Research Center, Mashhad Branch, Islamic Azad University, Mashhad, Iran

**Keywords:** Apoptosis, Angiogenesis, Zinc oxide nanoparticles, Flowcytometry, Antioxidant

## Abstract

**Background:**

Algae are one of the natural materials used to green synthesis of nanoparticles. This method leads to minimize the toxicity of the chemical materials used to nanoparticle synthesis.

**Methods:**

In this study, zinc oxide nanoparticles (ZnO NPs) synthesized by *Sargassum muticum* algae extraction used to evaluate its cytotoxicity and apoptotic properties on human liver cancer cell line (HepG2).

**Results:**

Trypan blue assay results demonstrate a concentration-dependent decrease in cell viability and MTT assay shows increased growth inhibition in time and dose-dependent manner. In addition, CAM assay confirmed the ability of ZnO NPs to inhibit angiogenesis, but chick morphology (both the CR and weight) was not changed. Apoptotic tests (annexin V/PI and AO/PI) show that green-synthesized ZnO NPs induce apoptosis in all three time points (24, 48 and 72h).

**Conclusions:**

Our results confirm the beneficial cytotoxic effects of green-synthesized ZnO NPs on Human liver cancer cell. This nanoparticle decreased angiogenesis and induces apoptosis, so we conclude that these nanoparticles can be used as a supplemental drug in cancer treatments.

## Background

With the advent and explosive development of nanotechnology in the 21st century, nanoparticles have been used to produce various products, which have led to the introduction of nanomaterials in various aspects of life such as industrial products, environmental materials, biomedical and agricultural industries (Zaman et al. 2014).

Zinc oxide nanoparticles (ZnO NPs) are the most important synthetic nanoparticle that due to their specific properties increasingly used in variety of products such as cosmetics, pigments, electronic equipment, bioimaging, drug delivery, wastewater treatment, and environmental remediation (Liu et al. 2016; Lee et al. 2013; Xiong 2013; Osmond and Mccall 2010; Hsu et al. 2013; Xiong et al. 2011). Increasing application of ZnO NPs in different areas may enhance the potential risk of human exposure; therefore, it seems absolutely necessary to investigate the cytotoxicity effects of ZnO NPs especially in mammalian cells.

It is suggested that nanomaterials are more toxic than their bulk counterparts. The mechanisms by which nanoparticles exerts their toxic effects are not well known, but some recent studies attributed it to their greater surface, permeability into cells, and accumulate within cells and organisms (Xiong et al. 2011), as well as, membrane damage, inflammation, DNA damage, apoptosis, and change in interactions between cells to cells and matrix (Abbasalipourkabir et al. 2015). Considerable research has been carried out to evaluate the cytotoxicity of nanoparticle in vivo and in vitro; in this regard, one of the most common recent mechanisms discussed in relation with ZnO NPs cytotoxicity is the increased production reactive oxygen species (ROS) (Guo et al. 2013). Overproduction of ROS by ZnO NPs, attributed to its small size, induces that DNA damage, cellular apoptosis, and activation of vital-signaling pathways include MAPK (Sharma et al. 2012).

An attractive and new method to reduce the toxicity of nanoparticles is to synthesis it with green method instead of the chemical method. In this approach, the plant components are used for synthesis nanoparticles instead of chemical agents for reducing and capping (Jha et al. 2009). Green synthesis is more eco-friendly, safe, simple, nontoxic, economical (low-priced), efficient, biocompatible, and water soluble than chemical and physical method (Salam et al. 2012; Kim et al. 2011; Bala et al. 2015).

A broad variety of plant extract are used for the biosynthesis of ZnO NPs including marine macroalgae *Sargassum muticum* (Azizi et al. 2014), *Citrus aurantifolia*, *Parthenium hysterophorus*, *Aloe vera* (Samat and Nor 2013; Rajiv et al. 2013; Gunalan et al. 2012), *Poncirus trifoliate* (Nagajyothi et al. 2013), *Corymbia citriodora* leaf extract (Zheng et al. 2015), *Ocimum basilicum* L. var. purpurascens (Salam et al. 2014), *Aloe vera* (Sangeetha et al. 2011), *Parthenium hysterophorus* (Rajiv et al. 2013), *Astragalus gummifer* (Darroudi et al. 2013), and *Sedum alfredii* (Qu et al. 2011).

In addition to the current usage of marine algae in human food production, fertilization, phycocolloids, and cosmetic ingredients; some researcher applied it for nanoparticles synthesis (Azizi et al. 2014; Sahayaraj et al. 2012; Prasad et al. 2013; Singaravelu et al. 2007; Senapati et al. 2012). *Sargassum muticum* is large brown seaweed (macroalga) of the class Phaeophyceae that native to the northwest Pacific region (Milledge et al. 2016). A wide variety of potentially bioactive compounds were identified in Sargassum species including sulphated polysaccharides fucoxanthin, steroids, terpenoids, and flavonoids (Milledge et al. 2016; Yende et al. 2014). *Sargassum muticum* maybe have some biochemical properties due to its unknown components (Yende et al. 2014; Pomin 2014). In our previous report, we used the aqueous extraction of *S. muticum* for synthesis the ZnO NPs by green method (Azizi et al. 2014); *S. muticum* has been used to make various nanoparticles, including zinc oxide nanoparticles (Azizi et al. 2014), Magnetic Iron Oxide (Fe_3_O_4_) Nanoparticles (Namvar and Mohamed 2016), and gold nanoparticles (Namvar et al. 2015). The human exposure to ZnO NPs is undeniable, and it established that major target organ for ZnO NPs is the liver. Therefore, the primary aims of the present study were to evaluate the cytotoxic effects of green-synthesized ZnO NPs on human liver cells (HepG2) and its antiangiogenic effect by CAM method.

## Methods

### Synthesis, characterization, SEM, and optical analysis of ZnO nanoparticles

The green synthesis of ZnO NPs was carried out according to a method described in our previous study. The data of characterization, SEM and optical analysis of ZnO NPs were published in our previous article (Azizi et al. 2014); in the current article, no relevant data are provided.

In brief, the mixture of 2 g dried powder of *S. muticum* algae and 100 ml distilled water heated up to 100 °C and then was filtered by Whatman 41 filter paper. 50 ml of the aqueous extract algae was mixed with 10 ml of 2 mM of zinc acetate di-hydrate solution and the mixture was stirred at 70 °C for 3–4 h in an aqueous bath system to complete the reaction. The product was collected and washed several times using the centrifuge (4000 × rpm for 10 min); then, the ZnO–S. Muticum was dried at 100 °C for 48 h, and finally, the purified green-synthesized ZnO NPs were obtained by heating at 450 °C for 4 h (Azizi et al. 2014).

### Cell culture and cytotoxicity of nZnO

The human liver cancer cell line (*HepG2*) was selected for the cytotoxicity study of green-synthesized ZnO NPs and obtained from the Iranian National Cell Bank (Pasteur Institute of Iran). Cells were cultured in RMPI 1640 medium (Sigma), pH 7.4, supplemented with 10% heat-inactivated fetal bovine serum (FBS) (Gibco), and 1% (w/v) antibiotic and antimycotic solution (Gibco) and incubated in a humidified atmosphere with 5% CO_2_ at 37 °C. The potential effects of ZnO NPs on viability of *HepG2* cells were analyzed by trypan blue dye and MTT assay.

### Trypan blue assay

This test was examined to determine the toxicity effects of green-synthesized ZnO NPs on viability of HepG2 cells. 100 μl/well medium containing HepG2 cells (5 × 103) were cultured, after 48 h cells were detached by trypsin. Equal volume of cells and trypan blue dye (10 μl) were mixed softly and the number of viable cells was counted.

### MTT assay

To carry out the MTT assay, the viable HepG2 cells with a concentration of 5 × 10^3^ (100 μl/well) were cultured in flat bottom 96-well plates for 24 h; then, the supernatant was discarded and replaced by 100 μl RPMI medium containing ZnO NPs (87, 175, 350, 700, 1400, and 2800 μg/ml). Experiments are done in triplicate for each incubation time (24, 48, and 72 h). At the end of the incubations, the MTT solution, at the final concentration 4 mg/ml in PBS, was added to each well and incubated for 4 h at 37 °C in a 5% CO_2_ atmosphere; then, the supernatant removed and replaced by DMSO (100 μl). At the end of assay, plate was read against a blank reagent at 570 nm after 10 min shaking. The cytotoxicity of samples on cells was expressed as IC_50_ which is concentration of the tested samples and reduces 50% of cellular growth than untreated control sample and was calculated using the following formula:

$${\text{Growth inhibition}} = {\text{OD control}}-{\text{OD treated sample}} \times 100/{\text{OD control}} .$$

### Apoptosis assay

#### Annexin V/propidium iodide

After seeding the HepG2 cells (1 × 10^6^ cells/well) in 6-well plates for 24 h, ZnO NPs were added to the cells in different final concentration for 24 h. We performed the following sequential action: (1) the cells detached with trypsin and EDTA; (2) centrifugation at 750×*g* for 5 min; (3) washing the pellet cells with PBS containing calcium; (4) cells were re-suspended in 100 μl binding buffer; (5) cells were mixed with 2 μl annexin V-FITC; and at the end, (6) 2 μl PI was added to mixture and the flow cytometric analysis was done.

#### Acridine orange/propidium iodide staining (AO/PI)

After 24 h incubation of 1 × 10^6^ cells/well of HepG2 cells in 6-well plates at 37 °C in a humidified CO_2_ incubator, treatment and control groups treated with 150 μg/ml ZnO NPs and 0.1% DMSO, respectively, for three timepoints (24, 48, and 72 h). Then, the cells were separated with trypsin and AO/PI dyes were added to suspension cells at an equal ratio (10 μl) and were examined under fluorescent microscope. All viable and early apoptotic cells only uptake the AO dye which bind to double-strand DNA and emit green fluorescence, while PI stained the necrotic and dead cells and look red.

#### Chick chorioallantoic membrane assay

Among the standard methods for evaluating the effects of agents on angiogenesis, we selected the chick chorioallantoic membrane (CAM) assay. Briefly, forty fertilized Ross chicken eggs, purchased from Toos company (Iran), were carefully cleaned with 70% alcohol and randomly divided into four groups (ten eggs in each group); group 1 was control (without any treatment); and three other groups were experimented (groups 2, 3, and 4 treated with concentrations of 50, 100, and 150 µg/ml ZnO NPs, respectively). Then, the eggs were inserted in an automatic rotation Incubator at 38–38.5 °C and 55–65% humidity. After 48 h of the incubation (chick embryo development period), a 1 cm^2^ window was made in the shell under laminar flow hood, next, the windows were sealed using sterile paraffin and lamellas, and then, the eggs were returned to incubator until day 8 and rotated manually twice a day. On the 8th day of incubation, each specified group was treated with proper concentration of ZnO NPs under aseptic condition, and then, the windows were resealed and the incubation continued for further 72 h. At the day 12 of incubation, the egg shells were softly removed and CAM were carefully separated and examined with and without microscope. The stereomicroscope instrument was used to taking photo for analyzing the number and length of blood vessels. The photo was analyzed by Image J program.

#### Antioxidant activity by the ABTS^+^ assay

Antioxidant effects of green-synthesized ZnO NPs were measured by the 2, 2′-azino-bis (3-ethyl benzthiazoline-6-sulfonic acid (ABTS) radical scavenging assay. To generation of ABTS radicals, the proportion ratio of 2:1 (v/v) of ABTS (7 mM) and potassium persulphate (2.45 mM) was mixed and kept at dark room temperature environment to completion of radical generation for 16 h. Then, water was used to preparation of interested dilution to adjust an absorbance of 0.756 at 734 nm. The concentration of diluted solution was 0.514 mM. Antioxidant activity of ZnO NPs was determined by adding 1 ml of different concentrations of ZnO NPs (175, 350, 700, 1400, and 2800 μg/ml) to 1 ml diluted solution (containing ABTS radicals), incubated at 37 °C for 1 h and read at 734 nm using a spectrophotometer. Standard of our assay was glutathione. The following formula was used to calculation of percentage of inhibition:

$${\text{Inhibition percentage }}\left( {\% {\text{IP}}} \right) = \left[ {\left( {{\text{Ac}}-{\text{As}}} \right)/{\text{Ac}}} \right] \times 100.$$

#### Statistical analysis

The Statistical Package for the Social Sciences (SPSS, V.18) was used to statistical analyses. The means of obtained data were analyzed using ANOVA and followed by Tukey HSD (honestly significant difference) test between groups. A *P* value of < 0.05 was used as the criterion for a statistically significant difference. All tests were performed triplicate and the results are expressed as mean values ± standard deviation (mean ± SD).

## Results

### Effect of ZnO NPs on viability

**Fig. 1:**
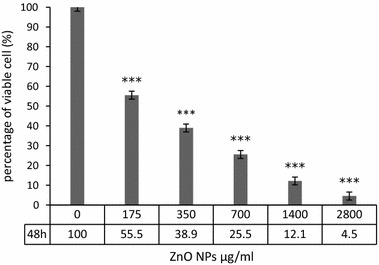
Trypan blue assay. Viability of HepG2 cell line was decreased on concentration-dependent manner in comparison with control group (***P value < 0.001)

The toxicity effects of green-synthesized ZnO NPs on viability of HepG2 cells were checked by MTT and Trypan blue assay. In trypan blue assay, the percent of viable HepG2 cells was calculated after 48 h incubation with different concentrations of ZnO NPs. The current research results demonstrate a concentration-dependent decrease in cell viability after 48 h exposure to ZnO NPs (Fig. 1). In the lowest concentration of ZnO NPs (175 μg/ml), more than half of cells remain survive (55.5%). In concentration more than 175 μg/ml, the viability of the cells was less than 40%. In the highest concentration of ZnO NPs, more than 95% of cells were dead and the survival rate of HepG2 cells was 4.5%.

### Effect of ZnO NPs on growth inhibition

**Fig. 2:**
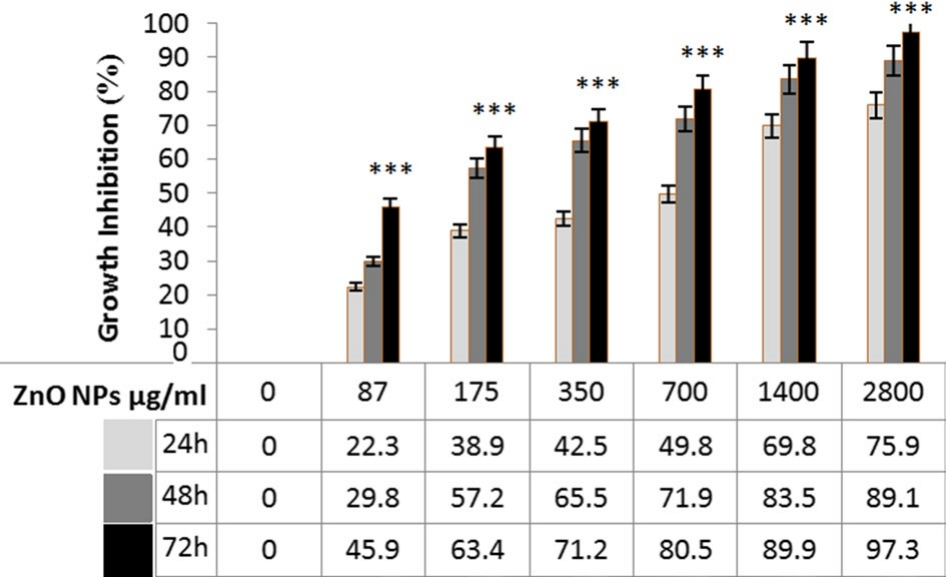
MTT assay. Growth inhibition effects of ZnO NPs on HepG2 in different times (24, 48, and 72 h) and concentration (87, 175, 350, 700, 1400, and 2800 μg/ml). The longer incubation time and increasing concentrations of nanoparticles lead to more percent inhibition of cell growth in comparison with control group (***P value < 0.001). All in vitro experiments were performed in triplicate and expressed ad the mean ± standard deviation

The growth inhibition of HepG2 cell was calculated in different concentrations (87, 175, 350, 700, 1400, and 2800 μg/ml) and times (24, 48, and 72 h) incubation with ZnO NPs by MTT assay (Fig. 2). The growth inhibition was increased in time- and dose-dependent manner. We observed that at the highest concentration of ZnO NPs (2800 μg/ml) after 72 h of exposure, more than 97% of HepG2 cells were died. The IC50 values were calculated 150 μg/ml at 48 h exposure time of ZnO NPs for both trypan blue and MTT assay. Our results show that green-synthesized ZnO NPs affected HepG2 cells viability even at low concentration.

### Acridine orange/propidium iodide

**Fig. 3:**
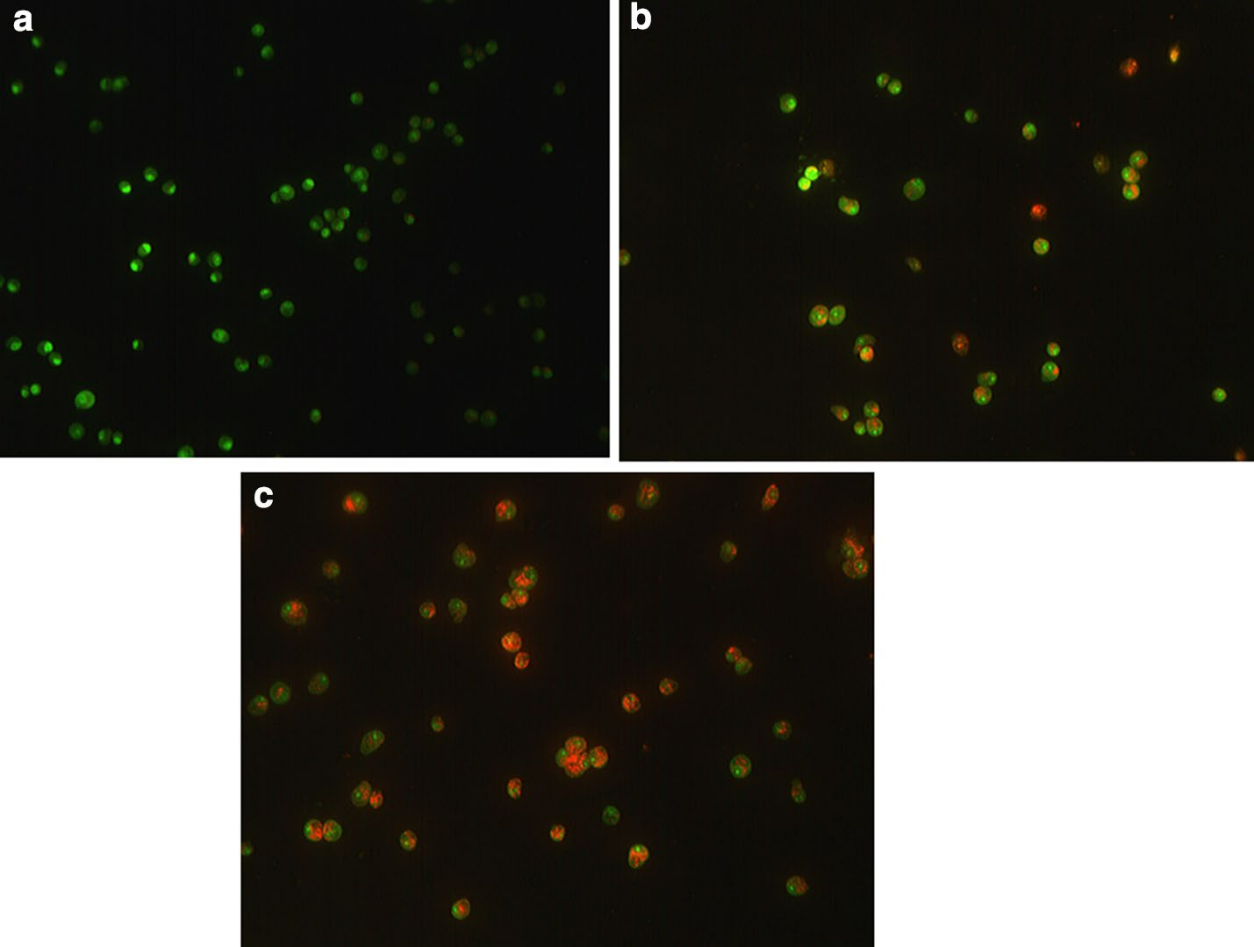
Fluorescent images of HepG2 cells stained by AO/PI. **a** control cells; **b** treat with 0.1% DMSO for 48 h; and **c** treated with 150 μg/ml ZnO NPs for 48 h (green: normal cancer cells, yellow: early apoptotic, and red: late apoptotic)

Table 1 and Fig. 3 present the results obtained from the AO/PI staining of HepG2 cells. From these data, we can see that ZnO NPs have time-dependent effects on cell viability. The results show decreased viability with higher apoptotic cells in all three timepoints (24, 48, and 72 h) of treatment with 0.1% DMSO and 150 μg/ml ZnO NPs. After 24 h treatment with DMSO viability of cells decreased to 93.7%, while, at the same time, the viability of ZnO NP treatment decreased to 70.9%. At 72 h, in DMSO treatment, the viability decreased to 87.3%, while, at the same time, the viability of ZnO NPs treatment decreased to 33.4%.

**Table 1:** Fluorescent results of morphological analysis of HepG2 cells using AO/PI staining

Group	Time	% Viable	% Apoptotic	% Necrotic
Control HepG2 + 0.1% DMSO	0	99 + 0.1	0.7 + 0.3	0.3 + 0.2
	24	93.7 ± 0.1	4.5 ± 1.7	1.8 ± 0.7
	48	92.3 ± 1.9	6.8 ± 3.2	2.9 ± 0.5
	72	87.3 ± 1.4	8.9 ± 2.4	4.1 ± 1.1
Treatment HepG2 + 150 μg/ml ZnO NPs	0	98.9 + 0.4	0.4 + 0.3	0.7 + 0.4
	24	70.9 ± 1	27.87 ± 6.6*	2.1 ± 0.9
	48	49.8 ± 1.6	43.5 ± 3.3*	6.7 ± 1.2
	72	33.4 ± 4.7	57.7 ± 2.7*	8.9 ± 2.5

All values are expressed as mean ± standard deviation. * *P* < 0.001

**Fig. 4:**
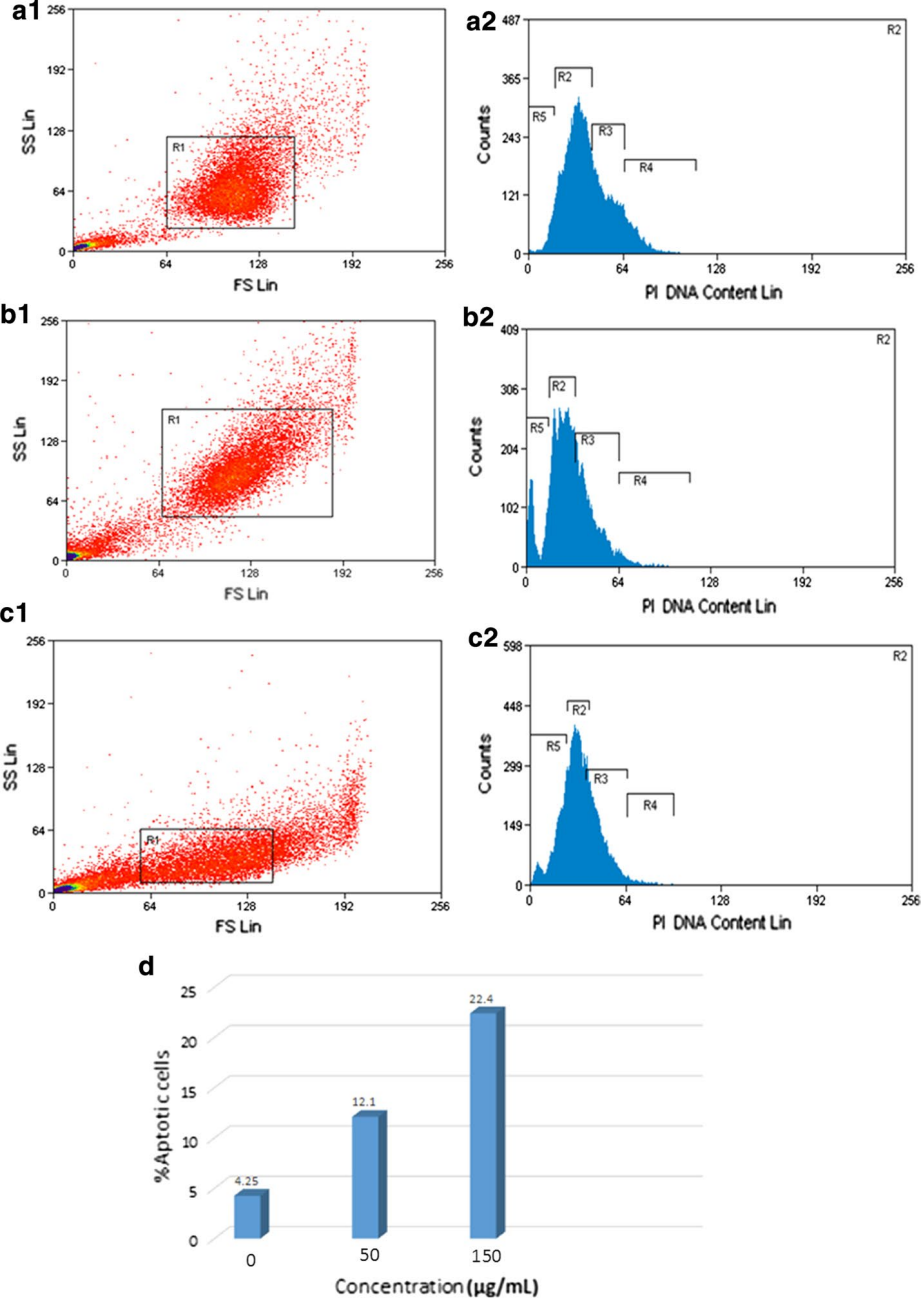
Apoptosis analysis in HepG2 cells by Annexin V-FITC/PI. (**a1**–**c1**): change in membrane asymmetry, and (**a2**–**c2**): analysis of cell cycle in HepG2 cells after 48 h incubation with ZnO NPs. **a** Untreated (control); **b** treated cells with 50 μg/ml ZnO NPs; and **c** treated cells with 150 μg/ml ZnO NPs. **d** Percentages of apoptosis HepG2 cells. R5: G0 that refers to the proportion of apoptotic cells

The apoptotic effects of green-synthesized ZnO NPs were investigated by annexin V-FITC/PI dyes using flow cytometric analysis. Using of these dyes determines the cell viability in the following different mode viable cells, early apoptosis cells, late apoptosis cells, and necrotic cells. The results, as shown in Fig. 4d, indicate that a higher concentration of ZnO NPs increases apoptotic cells.

### CAM assay

**Fig. 5:**
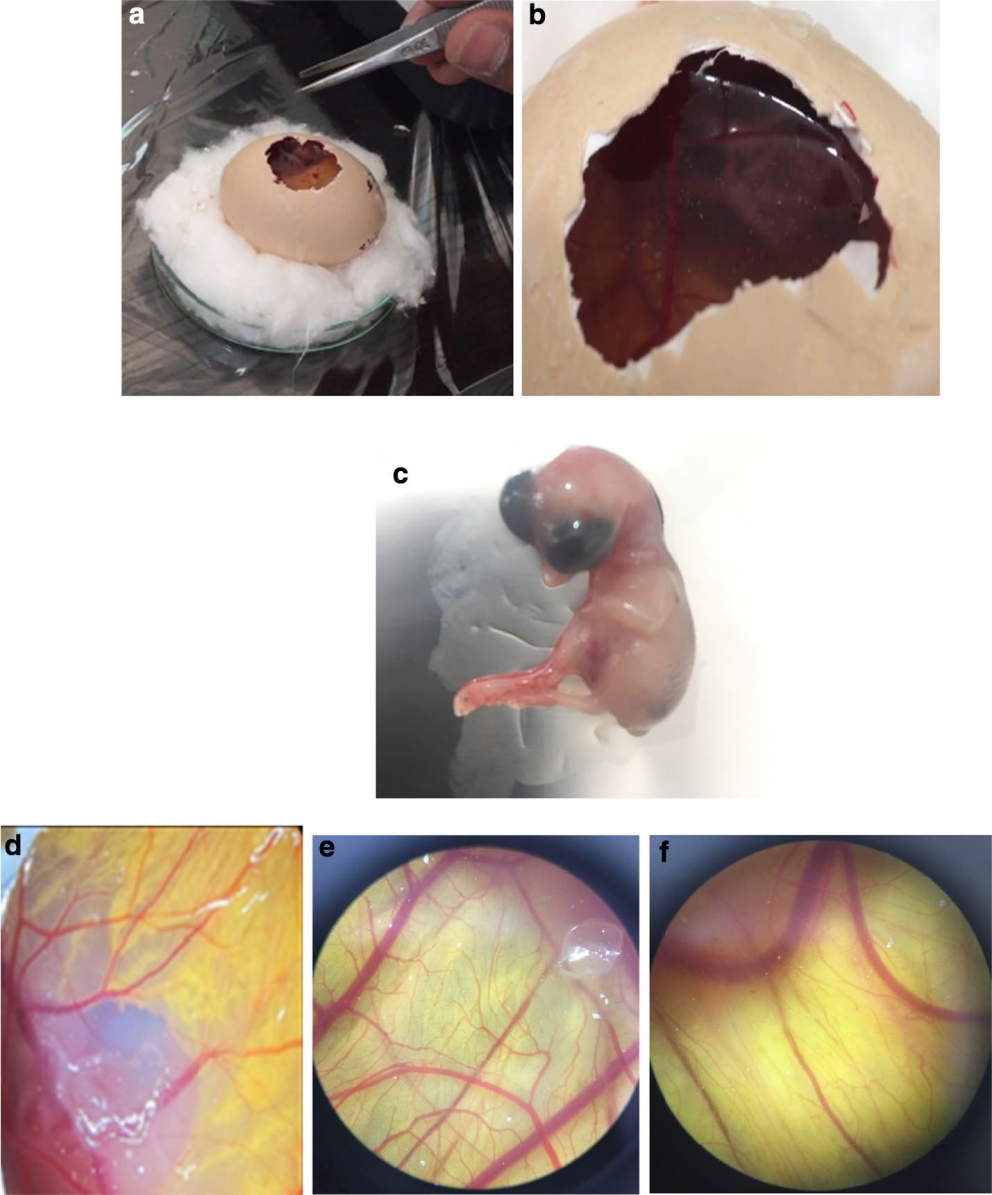
**a** Image of CAM with gelatin sponge and **b** square which counting was done there. **c** Control, **d** 50, **e** 100, and **f** 150 μg/ml concentrations of ZnO NPs

**Fig. 6:**
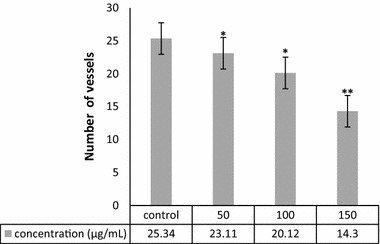
Comparison of number of new vessels in four groups. Treated groups compared with control group (*P < 0.05, **P < 0.001). Experiments performed in triplicate

**Fig. 7:**
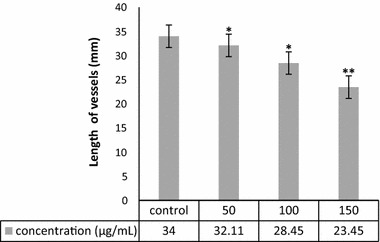
Comparison of length of new vessels in four groups. Treated groups compared with control group (*P < 0.05, **P < 0.001)

Angiogenesis was evaluated by CAM assay. The angiogenesis indicators (number and length of the newly formed arterioles) were not significantly decreased in control and treated groups with 50 and 100 μg/ml concentration of green-synthesized ZnO NPs. The eggs treated with higher concentration of ZnO NPs (150 μg/ml) showed significantly reduction of vessel formation (*P* < 0.001) (Figs. 5, 6, and 7). Based on the results from CAM model, ZnO NPs at the highest concentration demonstrated significant inhibition of angiogenesis as compared with the control group. At the end of study, no significant changes was observed in chick morphology (both the CR and weight) in control group and treated group with 150 μg/ml (data not shown).

### ABTS

**Fig. 8:**
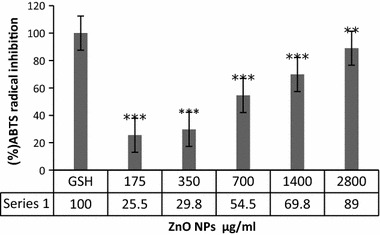
Radical inhibition activity of ZnO NPs. Treated groups compared with GSH group

Antioxidant activities of green-synthesized ZnO NPs evaluated by ABTS assay, relative to glutathione as standard group. Principle of this test based on quenching the long-lived ABTS radical cation (ABTS+) by antioxidant molecules. As shown in Fig. 8, the ABTS radical scavenging activities of green-synthesized ZnO NPs were dose-dependent and gradually increased. Maximum radical scavenging was observed up to 89% at concentration of 2800 μg/ml among different concentrations of 175, 350, 700, 1400, and 2800 μg/ml with EC50 600 μg/ml.

## Discussion

Despite advances in diagnosis and treatment of cancers, it is still the second leading cause of death in the word; liver cancer is the fifth leading cause of cancer-related deaths worldwide (Siegel et al. 2017). Recently, cancer treatment and diagnosis were improved using different nanoparticles as nanocarrier systems, in both in vitro and in vivo research.

Zinc oxide nanoparticle is one of the most interested nanoparticles applied as nanocarrier for cancer treatment as it is more cytotoxic to proliferative cells as compared to non-proliferative counterparts (Valdiglesias et al. 2013). The toxicological effects of chemical-based ZnO NPs have been investigated on wide variety of human cell lines include skin fibroblast (Dechsakulthorn et al. 2007) colon carcinoma LoVo cells (De Berardis et al. 2010), HepG2, A549, and BEAS-2B (Akhtar et al. 2012), A549 (Ahamed et al. 2011), and some primary rat cell lines (Akhtar et al. 2012), and a large number of other cells that not mentioned here. Akhtar et al. (2012) and Taccola et al. (2011) and a number of other researchers have noticed that ZnO NPs are cytotoxic only to cancerous cells and do not have any toxic effects on normal cell lines (Akhtar et al. 2012; Taccola et al. 2011). However, the cytotoxicity of some chemical synthesized nanoparticles has been confirmed on normal cells even at lower concentration in comparison of cancer cell lines; for this reason, green synthesis of nanoparticles becomes more attractive as an alternative method due to the decreasing biotoxicity effects of nanoparticles, through the use of non-noxious components instead of chemicals agent over the last recent years.

The most used natural material to green synthesis of ZnO NPs is made up of plant extraction (Salam et al. 2014; Fu and Fu 2015; Nethravathi et al. 2015), fungi (Jain et al. 2013; Kundu et al. 2014), bacteria (Jayaseelan et al. 2012), algae (Azizi et al. 2014), and natural agents such as honey (Hoseini et al. 2015). The brown seaweed, *S. muticum* described at introduction, is also a selective plant to synthesis of nanoparticles. The *S. muticum* has several components which provides antimicrobial, anticancer, antioxidant, antiinflammation (Khan et al. 2008), anti allergic (Zuercher et al. 2006), and anti diabetic properties (Perez et al. 1998).

This is assumed that nanoparticle synthesized by naturally components having high antioxidant activity is associated with more and more efficiency in reduction of toxicity in cells. Milledge et al. (2016) provided a description of potential use of seaweed in various disciplines (Milledge et al. 2016). Common antioxidant component found in *S. muticum* contain alpha and gamma tocopherol (Farvin and Jacobsen 2013), the carotenoid pigment fucoxanthin (Chae et al. 2013; Kumar et al. 2013; Wijesinghe and Jeon 2011), and other carotenoids and phenolic compounds (Milledge et al. 2016). In addition to antioxidant components, high amounts of fucoxanthin (as strong anticancer agent), carrageenan, alginates, iodine, agar, cytoprotection, phyco biliproteins, and many other important compounds such as essential fatty acids, sterols, and proteins result in pharmaceutical application of *S. muticum* (Milledge et al. 2016).

The toxic effects of methanolic extraction of *S. muticum* have investigated by (Namvar et al. 2013); they demonstrated that despite being it toxic to cancer cell lines (MCF-7 and MDA-MB-231 cell lines) is not toxic to normal Vero cell line. They suggested that specific inhibition of cancer cell growth caused by signaling pathways activated by ligand/receptor interaction. The ligands in natural material are probably presented at the surface of nanoparticles which can attach to specific receptors and trigger the downstream signaling cascade leading to cell death in cancer cells (Harada et al. 1997). Recent evidence suggests that polysaccharides are the primary component of seaweed having anticancer activity (Holdt and Kraan 2011).

Cytotoxicity of nanoparticles routinely went through by the trypan blue and MMT assay. In this study, we pointed out that ZnO NPs were found to cause reduction in HepG2 cells viability even at the lowest concentration by trypan blue assay. Green-synthesized ZnO NPs inhibits cell growth in a concentration-dependent manner (87–2800 μg/ml) leading to cell death in HepG2 cell line after 48 h incubation. The cytotoxic effect of ZnO NPs is clearly supported by the current findings of MTT assay. Time- and concentration-dependent decrease in cell viability was observed by MTT assay. In the present study, median lethal concentration (IC50) value of green-synthesized ZnO NPs was calculated 150 µg/ml at 48 h exposure. This finding is broadly in agreement with earlier studies (Dhamodarana and Kavithab 2015), which used the aqueous leaf extract of *Andrograohis Paniculata* to green synthesis of ZnO NPs. They observed the maximum anticancer activity of ZnO NPs against HeLa and Hep-2 cell lines at 250 µg/ml. Namvar et al. (2015) who demonstrated growth inhibitory effects of green-synthesized ZnO nanoparticles on 4T1, CRL- 1451, CT-26, and WEHI-3B cell lines by dose- and time-dependent manner (Namvar et al. 2015). They also showed that ZnO NPs have no cytotoxicity on normal mouse fibroblast (3T3) cell line (Namvar et al. 2015). This finding is in agreement with Valdiglesias et al. (2013) findings, who exhibited that exposure to chemical-based ZnO NPs significantly decreased the percentage of viable SH-SY5Y cells by dose-dependent manner at all interval times (Valdiglesias et al. 2013). Treatment with ZnO NPs led to reduction in viability of other cell lines, in a concentration-dependent manner, consists of A431 (epidermal carcinoma) (Sharma et al. 2009), U87 (astrocytoma) (Lai et al. 2008), HepG2 (human liver cancer) (Sharma et al. 2012), and A549 (lung carcinoma) (Fukui et al. 2012) cell lines. The results of Wahab et al. (2014) supported also the previous results that ZnO NPs decreased proliferative cell viability in concentration-dependent manner (Wahab et al. 2014).

In other study, Namvar and Mohamed (2016) used the aqueous extraction of *S. muticum* to synthesis the magnetic iron oxide (Fe_3_O_4_) nanoparticles. They confirmed its antimicrobial activities against six microorganisms (*Listeria monocytogenes*, *Escherichia coli,* and *Candida species*) (Namvar and Mohamed 2016); likewise, Namvar et al. (2014) confirmed anticancer activity of iron oxide nanoparticles against Jurkat cells, MCF-7 cells, and HepG2 cells (Namvar et al. 2014).

Induction of apoptosis and necrosis in HepG2 by ZnO NPs was investigated using dual-staining AO/PI. Table 1 compares the results obtained from the preliminary analysis of AO/PI staining. Green-synthesized ZnO NPs decrease HepG2 cell viability by apoptosis induction in time-dependent manner. As well as, Fig. 3 reveals that there are marked increases in cell death after 48 h incubation with ZnO NPs, under the fluorescence microscope. It can be seen from the data in Table 1 and Fig. 3 that ZnO NPs induces apoptosis in HepG2 cells. Subsequently, the flow cytometric method was done for analysis of apoptosis by dual-staining annexin V-FITC/PI. At the first stage of apoptosis process (early apoptosis), phosphatidyl serine (PS) translocated from inner to outer layers of plasma membrane; annexin V attached to PS with high affinity and makes it possible to detect early apoptosis in cells (Martin et al. 1995; Koopman et al. 1994). Figure 4 shows analysis of apoptosis in HepG2 cells treated with different concentrations of ZnO NPs. As can be seen from Fig. 4d, increasing percentages of apoptosis were observed in HepG2 cells. This finding confirms the cytotoxic effects of green-synthesized ZnO NPs on human liver cancer cell line (HepG2) by induction of apoptosis. The findings observed in this study mirror those of the previous studies that have examined the apoptotic effect of nanoparticles in different cell lines; Nagajyothi et al. (2016) showed that green-synthesized copper oxide nanoparticles induce apoptosis in human cervical carcinoma cells (HeLa cells); Kulandaivelu and Gothandam (2016) showed that biosynthesized silver nanoparticles increase apoptosis pathway in MCF-7 cell line (Kulandaivelu and Gothandam 2016). The study has also confirmed the findings of Namvar et al. (2014) which found that iron oxide nanoparticles induce cell cycle arrest and apoptosis in Jurkat cells, MCF-7 cells and HepG2 cells; similarly, Wahab et al. (2014) were found that ZnO NPs result in cell death by induction of apoptosis in HepG2 (liver cancer) and MCF-7 (breast cancer) cancer cells (Wahab et al. 2014).

These results agree with the findings of other studies, in which cell cycle arrest is the preliminary event of cell death triggered by ZnO NPs in wide variety of cell lines such as SHSY5Y cells and neuronal Schwann cells (RSC96) (Yin et al. 2012), MCF-7, and HepG2. However, the findings of Valdiglesias et al. (2013) study support the previous research and confirm that mitochondrial-independent pathways are involved in cell death induction by ZnO NPs (Valdiglesias et al. 2013).

Since the angiogenesis is a critical step in cancer development and metastasis, many efforts have been made to inhibit this process. Nanotechnology also has entered in this field, different nanoparticles synthesis and used to inhibit angiogenesis, but obtained results were varied. Both antiangiogenic and proangiogenic properties of nanoparticles have been seen in research. Mroczek-Sosnowska et al. (2015) showed that copper nanoparticles have proangiogenic effects that their results in line with the previous results of Kang et al. (Kang et al. 2011); they reported that polyvinylpyrrolidone-coated silver nanoparticles induce angiogenesis. However, in contrast to these findings, the majority of studies focused on the antiangiogenic effects of nanoparticles, gold NPs [133] (Kalishwaralal et al. 2011; Arvizo et al. 2011), Ag NPs (Gurunathan et al. 2009), perflurocarbon nanoparticles (Das et al. 2013), carbon allotropes (Grodzik et al. 2011), etc. Our results were in line with latest group of articles that nanoparticles inhibit angiogenesis. In the present study, green-synthesized ZnO NPs decreased angiogenesis in chick chorioallantoic membrane. ZnO NPs at the highest concentration demonstrated significant inhibition of angiogenesis as comparing with control group. The chick morphology had no significant changes.

## Conclusion

In this investigation, the aim was to assess the cytotoxic effects of ZnO NPs on HepG2 cells; as well as the antiangiogenic effects of ZnO NPs was evaluated. Current research results confirmed that ZnO NPs have apoptotic, antiangiogenic, and cytotoxic effects at all concentration and time of incubation. In general, therefore, it seems that it may be used as a supplemental drug in cancer treatment for decreasing angiogenesis and induce apoptosis. Finally, an important limitation needs to be considered that the current study has only examined in vitro toxicity tests methods for ZnO NPs; it is recommended that further research be undertaken in vivo.

## References

[CR1] Abbasalipourkabir R, Moradi H, Zarei S, Asadi S, Salehzadeh A, Ghafourikhosroshahi A, Mortazavi M, Ziamajidi N (2015). Toxicity of zinc oxide nanoparticles on adult male Wistar rats. Food Chem Toxicol.

[CR2] Ahamed M, Akhtar MJ, Raja M, Ahmad I, Siddiqui MKJ, AlSalhi MS, Alrokayan SA (2011). ZnO nanorod-induced apoptosis in human alveolar adenocarcinoma cells via p53, survivin and bax/bcl-2 pathways: role of oxidative stress. Nanomed Nanotechnol Biol Med.

[CR3] Akhtar MJ, Ahamed M, Kumar S, Khan MM, Ahmad J, Alrokayan SA (2012). Zinc oxide nanoparticles selectively induce apoptosis in human cancer cells through reactive oxygen species. Int J Nanomed.

[CR4] Arvizo RR, Rana S, Miranda OR, Bhattacharya R, Rotello VM, Mukherjee P (2011). Mechanism of anti-angiogenic property of gold nanoparticles: role of nanoparticle size and surface charge. Nanomed Nanotechnol Biol Med.

[CR5] Azizi S, Ahmad MB, Namvar F, Mohamad R (2014). Green biosynthesis and characterization of zinc oxide nanoparticles using brown marine macroalga *Sargassum muticum* aqueous extract. Mater Lett.

[CR6] Bala N, Saha S, Chakraborty M, Maiti M, Das S, Basu R, Nandy P (2015). Green synthesis of zinc oxide nanoparticles using Hibiscus subdariffa leaf extract: effect of temperature on synthesis, anti-bacterial activity and anti-diabetic activity. RSC Adv.

[CR7] Chae D, Manzoor Z, Kim SC, Kim S, Oh T-H, Yoo E-S, Kang H-K, Hyun J-W, Lee NH, Ko M-H (2013). Apo-9′-fucoxanthinone, isolated from *Sargassum muticum*, inhibits CpG-induced inflammatory response by attenuating the mitogen-activated protein kinase pathway. Marine drugs.

[CR8] Darroudi M, Sabouri Z, Oskuee RK, Zak AK, Kargar H, Hamid MHNA (2013). Sol–gel synthesis, characterization, and neurotoxicity effect of zinc oxide nanoparticles using gum tragacanth. Ceram Int.

[CR9] Das S, Schmieder A, Pan D, Senpan A, Caruthers S, Wickline S, Lanza G, Wagner E (2013). A nanoparticle based therapy to target bronchial angiogenesis. D108 lessons in angiogenesis across development and disease.

[CR10] De Berardis B, Civitelli G, Condello M, Lista P, Pozzi R, Arancia G, Meschini S (2010). Exposure to ZnO nanoparticles induces oxidative stress and cytotoxicity in human colon carcinoma cells. Toxicol Appl Pharmacol.

[CR11] Dechsakulthorn F, Hayes A, Bakand S, Joeng L, Winder C (2007). In vitro cytotoxicity assessment of selected nanoparticles using human skin fibroblasts. AATEX.

[CR12] Dhamodarana M, Kavithab S. Anticancer activity of zinc nanoparticles made using terpenoids from aqueous leaf extract of *Andrographis paniculata*. 2015.8(4):6

[CR13] Farvin KS, Jacobsen C (2013). Phenolic compounds and antioxidant activities of selected species of seaweeds from Danish coast. Food Chem.

[CR14] Fu L, Fu Z (2015). Plectranthus amboinicus leaf extract–assisted biosynthesis of ZnO nanoparticles and their photocatalytic activity. Ceram Int.

[CR15] Fukui H, Horie M, Endoh S, Kato H, Fujita K, Nishio K, Komaba LK, Maru J, Miyauhi A, Nakamura A (2012). Association of zinc ion release and oxidative stress induced by intratracheal instillation of ZnO nanoparticles to rat lung. Chem Biol Interact.

[CR16] Grodzik M, Sawosz E, Wierzbicki M, Orlowski P, Hotowy A, Niemiec T, Szmidt M, Mitura K, Chwalibog A (2011). Nanoparticles of carbon allotropes inhibit glioblastoma multiforme angiogenesis in ovo. Int J Nanomed.

[CR17] Gunalan S, Sivaraj R, Rajendran V (2012). Green synthesized ZnO nanoparticles against bacterial and fungal pathogens. Progress Nat Sci Mater Int.

[CR18] Guo D, Bi H, Liu B, Wu Q, Wang D, Cui Y (2013). Reactive oxygen species-induced cytotoxic effects of zinc oxide nanoparticles in rat retinal ganglion cells. Toxicol In Vitro.

[CR19] Gurunathan S, Lee K-J, Kalishwaralal K, Sheikpranbabu S, Vaidyanathan R, Eom SH (2009). Antiangiogenic properties of silver nanoparticles. Biomaterials.

[CR20] Harada H, Tadahide N, Kamei Y (1997). Selective antitumor activity in vitro from marine algae from Japan coasts. Biol Pharm Bull.

[CR21] Holdt SL, Kraan S (2011). Bioactive compounds in seaweed: functional food applications and legislation. J Appl Phycol.

[CR22] Hoseini SJ, Darroudi M, Oskuee RK, Gholami L, Zak AK (2015). Honey-based synthesis of ZnO nanopowders and their cytotoxicity effects. Adv Powder Technol.

[CR23] Hsu S-h, Lin YY, Huang S, Lem KW, Nguyen DH (2013). Synthesis of water-dispersible zinc oxide quantum dots with antibacterial activity and low cytotoxicity for cell labeling. Nanotechnology.

[CR24] Jain N, Bhargava A, Tarafdar JC, Singh SK, Panwar J (2013). A biomimetic approach towards synthesis of zinc oxide nanoparticles. Appl Microbiol Biotechnol.

[CR25] Jayaseelan C, Rahuman AA, Kirthi AV, Marimuthu S, Santhoshkumar T, Bagavan A, Gaurav K, Karthik L, Rao KB (2012). Novel microbial route to synthesize ZnO nanoparticles using *Aeromonas hydrophila* and their activity against pathogenic bacteria and fungi. Spectrochim Acta Part A Mol Biomol Spectrosc.

[CR26] Jha AK, Prasad K, Prasad K, Kulkarni A (2009). Plant system: nature’s nanofactory. Colloids Surf B.

[CR27] Kalishwaralal K, Sheikpranbabu S, BarathManiKanth S, Haribalaganesh R, Ramkumarpandian S, Gurunathan S (2011). RETRACTED ARTICLE: gold nanoparticles inhibit vascular endothelial growth factor-induced angiogenesis and vascular permeability via Src dependent pathway in retinal endothelial cells. Angiogenesis.

[CR28] Kang K, Lim D-H, Choi I-H, Kang T, Lee K, Moon E-Y, Yang Y, Lee M-S, Lim J-S (2011). Vascular tube formation and angiogenesis induced by polyvinylpyrrolidone-coated silver nanoparticles. Toxicol Lett.

[CR29] Khan MN, Choi JS, Lee MC, Kim E, Nam TJ, Fujii H, Hong YK (2008). Anti-inflammatory activities of methanol extracts from various seaweed species. J Environ Biol.

[CR30] Kim PS, Djazayeri S, Zeineldin R (2011). Novel nanotechnology approaches to diagnosis and therapy of ovarian cancer. Gynecol Oncol.

[CR31] Koopman G, Reutelingsperger C, Kuijten G, Keehnen R, Pals S, Van Oers M (1994). Annexin V for flow cytometric detection of phosphatidylserine expression on B cells undergoing apoptosis. Blood.

[CR32] Kulandaivelu B, Gothandam K. Cytotoxic effect on cancerous cell lines by biologically synthesized silver nanoparticles. Braz Arch Biol Technol. 2016;59:e16150529.

[CR33] Kumar SR, Hosokawa M, Miyashita K (2013). Fucoxanthin: A marine carotenoid exerting anti-cancer effects by affecting multiple mechanisms. Marine drugs..

[CR34] Kundu D, Hazra C, Chatterjee A, Chaudhari A, Mishra S (2014). Extracellular biosynthesis of zinc oxide nanoparticles using Rhodococcus pyridinivorans NT2: multifunctional textile finishing, biosafety evaluation and in vitro drug delivery in colon carcinoma. J Photochem Photobiol B.

[CR35] Lai JC, Lai MB, Jandhyam S, Dukhande VV, Bhushan A, Daniels CK, Leung SW (2008). Exposure to titanium dioxide and other metallic oxide nanoparticles induces cytotoxicity on human neural cells and fibroblasts. Int J Nanomed.

[CR36] Lee J, Choi S, Bae SJ, Yoon SM, Choi JS, Yoon M (2013). Visible light-sensitive APTES-bound ZnO nanowire toward a potent nanoinjector sensing biomolecules in a living cell. Nanoscale.

[CR37] Liu J, Feng X, Wei L, Chen L, Song B, Shao L (2016). The toxicology of ion-shedding zinc oxide nanoparticles. Crit Rev Toxicol.

[CR38] Martin S, Reutelingsperger CP, McGahon AJ, Rader JA, Schie RCv, LaFace DM, Green DR (1995). Early redistribution of plasma membrane phosphatidylserine is a general feature of apoptosis regardless of the initiating stimulus: inhibition by overexpression of Bc1-2 and Abl. J Exp Med.

[CR39] Milledge JJ, Nielsen BV, Bailey D (2016). High-value products from macroalgae: the potential uses of the invasive brown seaweed, *Sargassum muticum*. Rev Environ Sci Bio/Technol.

[CR40] Mroczek-Sosnowska N, Sawosz E, Vadalasetty KP, Lukasiewicz M, Niemiec J, Wierzbicki M, Kutwin M, Jaworski S, Chwalibog A (2015). Nanoparticles of copper stimulate angiogenesis at systemic and molecular level. Int J Mol Sci.

[CR41] Nagajyothi P, An TM, Sreekanth T, Lee JI, Lee DJ, Lee K (2013). Green route biosynthesis: characterization and catalytic activity of ZnO nanoparticles. Mater Lett.

[CR42] Nagajyothi P, Muthuraman P, Sreekanth T, Kim DH, Shim J (2016). Green synthesis: In-vitro anticancer activity of copper oxide nanoparticles against human cervical carcinoma cells. Arabian J Chem.

[CR43] Namvar F, Mohamed R. Biomedical application of green biosynthesis magnetic iron oxide (Fe_3_O_4_) nanoparticles using seaweed (*Sargassum muticum*) aqueous extract. Int J Chem Mol Eng. 2016;3(1). 10.3390/molecules18055954 PMC6270411 23698048

[CR44] Namvar F, Mohamad R, Baharara J, Zafar-Balanejad S, Fargahi F, Rahman HS. Antioxidant, antiproliferative, and antiangiogenesis effects of polyphenol-rich seaweed (*Sargassum muticum*). BioMed Res Int. 2013;2013:604787. 10.1155/2013/604787 PMC3776361 24078922

[CR45] Namvar F, Rahman HS, Mohamad R, Baharara J, Mahdavi M, Amini E, Chartrand MS, Yeap SK (2014). Cytotoxic effect of magnetic iron oxide nanoparticles synthesized via seaweed aqueous extract. Int J Nanomed.

[CR46] Namvar F, Azizi S, Ahmad MB, Shameli K, Mohamad R, Mahdavi M, Tahir PM (2015). Green synthesis and characterization of gold nanoparticles using the marine macroalgae *Sargassum muticum*. Res Chem Intermed.

[CR47] Namvar F, Rahman HS, Mohamad R, Azizi S, Tahir PM, Chartrand MS, Yeap SK (2015). Cytotoxic effects of biosynthesized zinc oxide nanoparticles on murine cell lines. EvidenceBased Complement Altern Med.

[CR48] Nethravathi P, Shruthi G, Suresh D, Nagabhushana H, Sharma S (2015). Garcinia xanthochymus mediated green synthesis of ZnO nanoparticles: photoluminescence, photocatalytic and antioxidant activity studies. Ceram Int.

[CR49] Osmond MJ, Mccall MJ (2010). Zinc oxide nanoparticles in modern sunscreens: an analysis of potential exposure and hazard. Nanotoxicology.

[CR50] Perez GR, Zavala SM, Perez GS, Perez GC (1998). Antidiabetic effect of compounds isolated from plants. Phytomedicine.

[CR51] Pomin VH (2014). Marine medicinal glycomics. Front Cell Infect Microbiol.

[CR52] Prasad TN, Kambala VSR, Naidu R (2013). Phyconanotechnology: synthesis of silver nanoparticles using brown marine algae *Cystophora moniliformis* and their characterisation. J Appl Phycol.

[CR53] Qu J, Luo C, Hou J (2011). Synthesis of ZnO nanoparticles from Zn-hyperaccumulator (*Sedum alfredii* Hance) plants. IET Micro Nano Lett.

[CR54] Rajiv P, Rajeshwari S, Venckatesh R (2013). Bio-fabrication of zinc oxide nanoparticles using leaf extract of *Parthenium hysterophorus* L. and its size-dependent antifungal activity against plant fungal pathogens. Spectrochim Acta Part A Mol Biomol Spectrosc.

[CR55] Sahayaraj K, Rajesh S, Rathi J (2012). Silver nanoparticles biosynthesis using marine alga Padina pavonica (Linn.) and its microbicidal activity. Digest J Nanomater Biostruct DJNB.

[CR56] Salam HA, Rajiv P, Kamaraj M, Jagadeeswaran P, Gunalan S, Sivaraj R (2012). Plants: green route for nanoparticle synthesis. Int Res J Biol Sci.

[CR57] Salam HA, Sivaraj R, Venckatesh R (2014). Green synthesis and characterization of zinc oxide nanoparticles from *Ocimum basilicum* L. var. purpurascens Benth.-Lamiaceae leaf extract. Mater Lett.

[CR58] Samat NA, Nor RM (2013). Sol–gel synthesis of zinc oxide nanoparticles using *Citrus aurantifolia* extracts. Ceram Int.

[CR59] Sangeetha G, Rajeshwari S, Venckatesh R (2011). Green synthesis of zinc oxide nanoparticles by aloe barbadensis miller leaf extract: structure and optical properties. Mater Res Bull.

[CR60] Senapati S, Syed A, Moeez S, Kumar A, Ahmad A (2012). Intracellular synthesis of gold nanoparticles using alga *Tetraselmis kochinensis*. Mater Lett.

[CR61] Sharma V, Shukla RK, Saxena N, Parmar D, Das M, Dhawan A (2009). DNA damaging potential of zinc oxide nanoparticles in human epidermal cells. Toxicol Lett.

[CR62] Sharma V, Anderson D, Dhawan A (2012). Zinc oxide nanoparticles induce oxidative DNA damage and ROS-triggered mitochondria mediated apoptosis in human liver cells (HepG2). Apoptosis.

[CR63] Siegel RL, Miller KD, Jemal A (2017). Cancer statistics, 2017. CA Cancer J Clin.

[CR64] Singaravelu G, Arockiamary J, Kumar VG, Govindaraju K (2007). A novel extracellular synthesis of monodisperse gold nanoparticles using marine alga, *Sargassum wightii* Greville. Colloids Surf B.

[CR65] Taccola L, Raffa V, Riggio C, Vittorio O, Iorio MC, Vanacore R, Pietrabissa A, Cuschieri A (2011). Zinc oxide nanoparticles as selective killers of proliferating cells. Int J Nanomed.

[CR66] Valdiglesias V, Costa C, Kiliç G, Costa S, Pásaro E, Laffon B, Teixeira JP (2013). Neuronal cytotoxicity and genotoxicity induced by zinc oxide nanoparticles. Environ Int.

[CR67] Wahab R, Siddiqui MA, Saquib Q, Dwivedi S, Ahmad J, Musarrat J, Al-Khedhairy AA, Shin H-S (2014). ZnO nanoparticles induced oxidative stress and apoptosis in HepG2 and MCF-7 cancer cells and their antibacterial activity. Colloids Surf B.

[CR68] Wijesinghe W, Jeon Y-J (2011). Biological activities and potential cosmeceutical applications of bioactive components from brown seaweeds: a review. Phytochem Rev.

[CR69] Xiong HM (2013). ZnO nanoparticles applied to bioimaging and drug delivery. Adv Mater.

[CR70] Xiong D, Fang T, Yu L, Sima X, Zhu W (2011). Effects of nano-scale TiO 2, ZnO and their bulk counterparts on zebrafish: acute toxicity, oxidative stress and oxidative damage. Sci Total Environ.

[CR71] Yende SR, Harle UN, Chaugule BB (2014). Therapeutic potential and health benefits of *Sargassum* species. Pharm Rev.

[CR72] Yin Y, Lin Q, Sun H, Chen D, Wu Q, Chen X, Li S (2012). Cytotoxic effects of ZnO hierarchical architectures on RSC96 Schwann cells. Nanoscale Res Lett.

[CR73] Zaman M, Ahmad E, Qadeer A, Rabbani G, Khan RH (2014). Nanoparticles in relation to peptide and protein aggregation. Int J Nanomed.

[CR74] Zheng Y, Fu L, Han F, Wang A, Cai W, Yu J, Yang J, Peng F (2015). Green biosynthesis and characterization of zinc oxide nanoparticles using *Corymbia citriodora* leaf extract and their photocatalytic activity. Green Chem Lett Rev.

[CR75] Zuercher AW, Fritsche R, Corthésy B, Mercenier A (2006). Food products and allergy development, prevention and treatment. Curr Opin Biotechnol.

